# Assessing the measurement properties of PROMIS Computer Adaptive Tests, short forms and legacy patient reported outcome measures in patients undergoing total hip arthroplasty

**DOI:** 10.1186/s41687-024-00799-5

**Published:** 2024-10-21

**Authors:** C. Braaksma, N. Wolterbeek, M. R. Veen, R. W. Poolman, Y. Pronk, A. D. Klaassen, R. W. J. G. Ostelo, C. B. Terwee

**Affiliations:** 1 St. Antonius Hospital, Utrecht, The Netherlands; 2 OLVG, Amsterdam, The Netherlands; 3 Leiden University, Leiden, The Netherlands; 4 Research Department, Kliniek ViaSana, Mill, The Netherlands; 5 Department of Health Sciences, Faculty of Science, Amsterdam Movement Sciences, Vrije Universiteit Amsterdam, Amsterdam, The Netherlands; 6 Department of Epidemiology and Data Science, Amsterdam UMC Location Vrije Universiteit, Amsterdam, The Netherlands; 7 Amsterdam Public Health Research Institute, Methodology, Amsterdam, The Netherlands; 8 Department of Orthopaedic Surgery, St. Antonius Hospital Utrecht, P.O. Box 2500, Nieuwegein, 3430 EM The Netherlands

**Keywords:** Outcome measures, PROMs, Computer Adaptive Testing, PROMIS, Total hip arthroplasty

## Abstract

**Background:**

The commonly used (‘legacy’) PROMs evaluating outcomes of total hip arthroplasty (THA), have several limitations regarding their measurement properties and interpretation of scores. One innovation in PROMs is the use of Computerized Adaptive Testing (CAT). The Patient-Reported Outcomes Measurement Information System (PROMIS^®^) is a validated system of CATs. The aim of this study was to assess the measurement properties of PROMIS and legacy instruments in patients undergoing THA.

**Methodology:**

Patients in this multicenter study filled out a questionnaire twice, including Dutch-Flemish PROMIS v1.2 Physical Function (PROMIS-PF) and v1.1 Pain Interference (PROMIS-PI) CATs and short forms, PROMIS v1.0 Pain Intensity, and legacy PROMs (Hip disability and Osteoarthritis Outcome Score (HOOS), HOOS-Physical function Shortform (HOOS-PS), Western Ontario and McMaster Universities Osteoarthritis Index (WOMAC), Oxford Hip Score (OHS), and two numeric rating scales measuring pain). The reliability, measurement precision (Standard Error of Measurement (SEM)), smallest detectable change (SDC), and burden of PROMIS instruments were presented head-to-head to legacy PROMs. Furthermore, construct validity was assessed.

**Results:**

208 patients were included. All instruments had a sufficient test-retest reliability (range ICC: 0.83–0.96). The SEM of PROMIS CATs and short forms ranged from 1.8 to 2.2 T-score points, the SEM of legacy instruments 2.6–11.1. The SDC of PROMIS instruments ranged from 2.1 to 7.3 T-score points, the SDC of legacy instruments 7.2–30.9. The construct validity of PROMIS CAT and short forms were found sufficient, except for the PROMIS-PI short form. The burden of PROMIS CATs was smaller than PROMIS short forms (range 4.8–5.2 versus 8–20 items, respectively). The burden of legacy instruments measuring physical functioning ranged from 5 to 40 items.

**Conclusions:**

The PROMIS-PF is less burdensome, with high measurement precision, and almost no minimal or maximal scores, and an equal reliability compared to legacy instruments measuring physical functioning in patients undergoing THA. The PROMIS Pain Intensity 1a is comparable to the legacy pain instruments in terms of burden, reliability and SDC. Measuring the construct Pain Interference may not have additional value in this population because of its high correlation with instruments measuring physical functioning. The SDC values presented in this study can be used for individual patient monitoring.

**Supplementary Information:**

The online version contains supplementary material available at 10.1186/s41687-024-00799-5.

## Background

Patient reported outcome measures (PROMs) are questionnaires, aiming to obtain information about perceived symptoms and functioning of the patient. PROMs are increasingly used in clinical practice to screen and monitor patient’s symptoms and functioning, to facilitate informed and shared-decision making, and to improve quality-of-care [18]. However, much is still unknown about the optimal application of PROMs in daily clinical practice.

Total hip arthroplasty (THA) is number four ranked most frequently performed inpatient surgical procedure in the USA [26]. The use of PROMs in healthcare requires reliable and valid PROMs, with as little burden as possible for the patient. Unfortunately, the commonly used (called ‘legacy’) PROMs evaluating outcomes of THA, have several limitations regarding their measurement properties and interpretation of scores [5, 8, 19, 28]. For example, the measurement error is often too large for reliable use of PROMs for individual patients, questions are often not relevant for all patients or not at all time points, and there is a lack of responsiveness, thereby hampering the ability to measure treatment effects [5, 8, 28]. Lastly, many of these PROMs have a limited measurement range causing floor and ceiling effects [5, 8, 28]. In conclusion, the legacy PROMs are not optimal for individual clinical assessment.

One promising innovation in PROMs is the use of Computerized Adaptive Testing (CAT). CAT can be used with PROMs that are developed using Item Response Theory (IRT) modelling [15]. IRT item banks are large sets of questions that are ordered in terms of their difficulty on an underlying metric. Using CAT, the most informative questions from item banks are selected depending on previous answers given by patients, until a predefined reliability is reached [4]. Patients are more likely to answer only relevant questions because e.g., questions about running will not be asked if a patient answers that he has difficulty walking one mile. Patients need to complete on average only four to seven questions to get a reliable score [37]. The use of CAT will decrease patient burden and, since the item banks cover the full width of the domain, floor and ceiling effects are less likely. The Patient-Reported Outcomes Measurement Information System (PROMIS^®^) is the most carefully developed and extensively validated system of CATs for measuring health outcomes [7], and is increasingly used in orthopedic clinical practice [2, 6, 21, 25, 32, 34]. In addition to CAT, all PROMIS measures are also available as static short forms, containing a fixed subset of questions from the item bank. The short form scores are expressed on the same metric (scale) as scores obtained through CAT, and therefore, directly comparable. These short forms could be administered when CAT is not (yet) technically possible within the data collection system of a clinic.

Using a PROM in individual clinical care is only helpful when the clinician and patient can interpret the score, and more specifically the change score over time. If the clinician or the patient is interested if a change in score is a real change (not due to measurement error), it is important that the Smallest Detectable Change (SDC) of the measurement instrument is known. The SDC is defined as the smallest change that can be detected by the instrument, beyond measurement error. There is little published data regarding the smallest detectable change (SDC) of PROMIS Physical Function or Pain Interference in the orthopedic field [36]. Furthermore, the theoretical benefit of PROMIS CAT and short forms administering patient friendly and relevant questionnaires, need to be confirmed in the clinical setting. Therefore, measurement properties of PROMIS CAT and short forms have to be determined presented head-to-head with the legacy PROMs in patients undergoing arthroplasty to investigate if PROMIS CAT and short forms overcome the limitations of the legacy PROMs.

The aim of this study was to assess and present the reliability, measurement precision, smallest detectable change, and burden of the Dutch-Flemish PROMIS Physical Function and Pain Interference CATs and short forms, and PROMIS Pain intensity head-to-head to legacy PROMs (the Hip disability and Osteoarthritis Outcome Score (HOOS), the HOOS-Physical function Shortform (HOOS-PS), Western Ontario and McMaster Universities Osteoarthritis Index (WOMAC v3.1), Oxford Hip Score (OHS), and two numeric rating scales measuring pain at rest and pain during activity) in patients undergoing THA. Furthermore, construct validity of PROMIS CATs and short forms was assessed.

## Methods

The study involved three orthopedic departments with high volumes of THAs in the Netherlands (St. Antonius Hospital Utrecht, Kliniek ViaSana Mill, OLVG Amsterdam). The study was conducted according to the principles of the Declaration of Helsinki. The study was reviewed by a Medical Ethics Review Committee (MEC-U) (St. Antonius Hospital, Nieuwegein, the Netherlands) (W21.037), which confirmed that the Medical Research Involving Human Subjects Act (WMO) does not apply. With this waiver, approval of the Institutional Review Board of each participating center was obtained.

### Study participants

To ensure variability in PROM scores and to increase generalizability of the study results, two cohorts of patients were asked to participate: (1) patients currently on the waiting list for a THA and (2) patients who already underwent surgery. The patients in the second cohort were included at 3, 6 or 12 months post-surgery. As a rule of thumb, a sample size of 100 is considered as very good for the assessment of measurement properties [31]. To be eligible, patients had to be 18 years or older, and on the waiting list for a primary THA or 3, 6 or 12 months post-surgery. Exclusion criteria were THA for femoral neck fracture, patients unable to independently fill out questionnaires, insufficient knowledge of the Dutch language, or no internet facilities. Furthermore, patients who had surgery between test and retest were excluded. If patients were eligible and willing to participate, they were asked to sign the informed consent form digitally using an online informed consent module. Each hospital included a minimum of 25 patients, distributed over the measurement points.

### Procedure

Patients were asked to fill out an online questionnaire twice within a two-week interval through a web-based platform (OnlinePROMS, Interactive Studios, ‘s-Hertogenbosch, the Netherlands). This is a certified (ISO27001; NEN7510), online PROMs platform, which is linked to the CAT software of the Dutch-Flemish Assessment Center, part of the Dutch-Flemish PROMIS National Center. A two-week interval was chosen to ensure no (large) changes in pain and function, which is a design requirement for assessing reliability, including smallest detectable change. A maximum of two automatic reminders were sent every two days after the first invitation when the patient had not responded. After two reminders the patient was considered lost-to-follow-up.

### Measures

The questionnaire included two Dutch-Flemish PROMIS CATs, five Dutch-Flemish PROMIS short forms, one single PROMIS pain item, and six legacy PROMs. The retest questionnaire included the same questionnaires. The online platform did not allow for any missing values within questionnaires. Two PROMIS CAT measures were included: PROMIS v1.2 CAT Physical Function (PROMIS-PF) and PROMIS v1.1 CAT Pain Interference (PROMIS-PI; Table 1). The PROMIS CATs use a T-score metric with a mean of 50 and SD of 10, where 50 represents the mean score of the general population. A higher PROMIS T-score represents more of the concept being measured (i.e. better function or more pain). The items in the CAT were selected based on their statistical ability to best further refine the individual’s score, estimated from the already administered items. The CATs were automatically stopped when a Standard Error (SE) of 2.2 (95% reliability) was reached or a maximum of 12 items was administered. The CAT software used a Maximal Likelihood estimation (which was experimentally used for a while in the Netherlands with permission from HealthMeasures), in which in absence of variation in answer patterns, the calculation of the T-score and SE could were imputated (in this study the assigned scores were 0 or 100). Whenever a score could not be calculated using the ML estimation, the output of the score was 0 or 100 and registered as a minimum or maximum score. Table 2 shows the number and percentage of the patients with a minimum or maximum score.

**Table 1:** Characteristics of included measurement instruments

Questionnaire	Construct/definition	Items	Response options	Score	Recall	Reference
**PROMIS measures**						
PROMIS CAT Physical Function (PROMIS-PF, v1.2)	Functioning of one’s upper extremities (dexterity), lower extremities (walking or mobility), and central regions (neck, back), as well as instrumental activities of daily living	Min 3 Max 12	5-point Likert	T-score^1^	–	[11, 12]
PROMIS CAT Pain Interference (PROMIS-PI, v1.1)	Consequences of pain on relevant aspects of one’s life	Min 3 Max 12	5-point Likert	T-score^1^	Last 7 days	[35]
PROMIS Physical Function SF8b (v1.2)	Functioning of one’s upper extremities (dexterity), lower extremities (walking or mobility), and central regions (neck, back), as well as instrumental activities of daily living	8	5-point Likert	T-score^1^	–	[33, 38]
PROMIS Physical Function SF10a (v1.2)	Functioning of one’s upper extremities (dexterity), lower extremities (walking or mobility), and central regions (neck, back), as well as instrumental activities of daily living	10	5-point Likert	T-score^1^	–	[33, 38]
PROMIS Physical Function SF20a (v1.2)	Functioning of one’s upper extremities (dexterity), lower extremities (walking or mobility), and central regions (neck, back), as well as instrumental activities of daily living	20	5-point Likert	T-score^1^	–	[33, 38]
PROMIS Pain Interference SF8a (v1.1)	Consequences of pain on relevant aspects of one’s life	8	5-point Likert	T-score ^1^	Last 7 days	[1, 10]
PROMIS Pain Intensity 1a (v1.0)	How much a person hurts	1	11-option numeric rating scale	0 (no pain) − 10 (worst thinkable pain)	Last 7 days	[20, 30]
**Disease specific legacy PROMs**						
Hip disability and Osteoarthritis Outcome Score (HOOS)	5 subscales • Pain • Symptoms • Stiffness • Function in daily living (ADL) • Function in sport and recreation (Sport/Rec) • Hip related Quality of Life (QOL)	40 • 10 • 3 • 2 • 17 • 4 • 4	5-point Likert	0 (indicating extreme symptoms)–100 (indicating no symptoms)	Last week	[23]
HOOS- Physical Function Short form (HOOS-PS)	Physical functioning	5	5-point Likert	Raw scores were converted (0–100, 0 indicating extreme symptoms) [9]	Last week	[13]
Western Ontario and McMaster Universities Osteoarthritis Index (WOMAC)	3 subscales: • Pain • Stiffness • Function	24 • 5 • 2 • 17	5-point Likert	Raw scores were converted (0–100, 0 indicating extreme symptoms) [9]	Last 48 h	[3]
Oxford Hip Score (OHS)	function and pain	12	5-point Likert	0–48 (0 indicating the worst, 48 the best outcome)	Past 4 weeks	[14]
NRS Pain activity	Pain during activity	1	11-option numeric rating scale	0–100 (0 indicating the worst, 100 the best outcome)	Last week	No reference available
NRS Pain rest	Pain at rest	1	11-option numeric rating scale	0–100 (0 indicating the worst, 100 the best outcome)	Last week	No reference available

^1^T-score 50 represents the average score of the general population, SD of 10

**Table 2:** The ICC, the mean SEM, SDC and burden, the percentage patients with minimum and maximum scores and the range of PROMIS CAT, PROMIS short forms and legacy instruments (*n* = 208)

	ICC agreement (CI)	SEM mean (range)	SDC mean (range)	Burden (mean) number of items	Minimum score (%)	Maximum score (%)	Score range
PROMIS-PF	0.91 (0.88–93)	2.2 (1.7–3.5)	6.9 (4.7–9.6)	5.2	0%	0.7%	20.3–74.1
PROMIS-PI	0.91 (0.87–0.93)	2.1 (1.9–5.9)	6.8 (5.2–16.4)	4.8	13.2%	0.2%	44.6–76.8
PROMIS PF SF8b	0.96 (0.95–0.97)	2.2 (1.5–5.9)	5.1 (4.2–16.4)	8	0%	0%	20.9–59.7
PROMIS PF SF10a	0.93 (0.91–0.95)	2.2 (1.7–5.9)	6.6 (4.7–16.4)	10	0%	0%	20.9–61.9
PROMIS PF SF20a	0.95 (0.94–0.96)	1.8 (1.3–5.7)	5.5 (3.6–15.8)	20	0%	0%	20.6–62.7
PROMIS PI SF8a	0.94 (0.92–0.95)	2.4 (1.3–5.9)	7.3 (3.6–16.4)	8	0%	0%	40.7–77
PROMIS Pain Intensity 1a	0.95 (0.93–0.96)	0.8	2.1	1	18.3%	0.5%	0–10

	ICC agreement (CI)	SEM	SDC	Burden number of items	Minimum score (%)	Maximum score (%)	Score range
HOOS-PS	0.83 (0.78–0.88)	9.7	26.9	5	9.6%	0.5%	0–100
HOOS	0.95 (0.93–0.96)	6.3	17.6	40	0%	2.2%	1.9–100
HOOS-Symptoms	0.91 (0.89–0.93)	8.3	22.9	5	0.2%	9.8%	0–100
HOOS-QOL	0.95 (0.93–0.96)	7.5	20.9	4	9.4%	9.1%	0–100
HOOS-Sport/Recr	0.88 (0.84–0.91)	11.1	30.9	4	5.7%	7.4%	0–100
HOOS-ADL	0.92 (0.89–0.94)	7.5	20.7	17	0%	6.7%	1.5–100
HOOS-Pain	0.93 (0.91–0.95)	7.9	22	10	0.2%	15.3%	0–100
OHS	0.96 (0.94–0.97)	2.6	7.2	12	0%	7.2%	5–48
WOMAC	0.92 (0.90–0.94)	7.6	21	24	0%	5.4%	3.1–100
WOMAC—pain	0.90 (0.87–0.92)	9.1	25.2	5	0.5%	22%	0-100
WOMAC—Stiffness	0.87 (0.83–0.90)	10.5	29.2	2	2.9%	13.2%	0–100
WOMAC—Function	0.92 (0.89–94)	7.9	22	17	0%	6.7%	1.5–100
NRS pain Activity	0.93 (0.91–95)	9.2	25.4	1	18.6%	2.2%	0–100
NRS pain Rest	0.92 (0.90–0.94)	8.5	23.6	1	29.4%	0.1%	0–100

*Abbreviations ICC* intra-class correlation coefficient, *SDC* smallest detectable change, *SEM* standard error of measurement, *CI* confidence interval, *QOL* quality of life, *Sport/Recr* sports/recreation, *ADL* activities of daily living, *NRS* numeric rating scale, *PI* pain interference, *PF* physical function

Moreover, PROMIS short forms were administered: one measuring Pain Interference (SF8a) and three measuring Physical Function (SF8b, SF10a, and SF20a). These short forms contain a fixed set of items (Table 1). Scores are expressed on the same metric (scale) as scores obtained through CAT and, therefore, directly comparable. Furthermore, the PROMIS v1.0 Pain Intensity item 1a (also called Global07) was included. This item is also included in the PROMIS v1.2 Scale Global Health, validated as a brief measure of health related quality of life [20, 30]. Moreover, the questionnaire included disease specific legacy PROMs: the Hip disability and Osteoarthritis Outcome Score (HOOS), the HOOS-Physical function Shortform (HOOS-PS), Western Ontario and McMaster Universities Osteoarthritis Index (WOMAC v3.1), Oxford Hip Score (OHS), and two numeric rating scales measuring pain at rest and pain during activity (Table 1). The HOOS-PS and WOMAC were derived from the HOOS. Finally, the questionnaire included demographic and clinical characteristics (e.g. sex, age, joint, side, date of surgery).

### Outcomes

#### Reliability

##### Test-retest reliability

The test-retest reliability of the PROMIS CATs, PROMIS short forms and the legacy instruments was assessed by calculating the intra-class correlation coefficient (ICC) for each total- and/or subscale separately. Patients were invited twice within a two-week interval and, therefore, considered stable.

##### Measurement precision

The Standard Error of Measurement (SEM) at one time point was calculated as a parameter of measurement precision. PROMIS CATs and short forms were developed under an IRT model, in which each T-score is associated with its own standard error of measurement (SEM = SE(T-score)). The measurement error differs across the scale, each score (thus each patient) has its own SEM value. The legacy PROMs were developed under a Classical Test Theory (CTT) model, which assumes that all scores have the same SEM, so each PROM has one SEM value.

##### Smallest detectable change

Not every change on a measurement instrument can be considered a ‘real’ change. Small changes may be due to measurement error. The test-retest data were used to calculate the smallest detectable change (SDC), which is the smallest change in score that can be considered a ‘real’ change, above measurement error. The SDC is defined as the amount of change above which there is at least 95% chance that a real change has occurred [16].

#### Validity

##### Construct validity

Construct validity is defined as the degree to which the scores are consistent with hypotheses based on the assumption that the PROM validly measures the construct to be measured [27]. Hypotheses were formulated a priori about the expected correlations between the PROMIS CAT and PROMIS short forms with the comparator legacy instruments per measured domain. Correlations with measurement instruments measuring the same construct (e.g. PROMIS-PF and the HOOS-PS) were expected to be strong. Also, the PROMIS CAT and SF Pain Interference should highly correlate with comparator instruments measuring physical functioning, according to previous research in patients with musculoskeletal conditions and pain (e.g. in patients with chronic pain [11], spinal pain [22], and foot and ankle conditions [29]). This is expected because when pain levels increase, an individual’s physical function decreases. Furthermore, the correlations with measurements instruments measuring the same construct, should be higher than measurement instruments measuring different but related constructs (e.g. PROMIS-PF and legacy measures of pain, stiffness or quality of life).

#### Interpretability

##### Burden

The number of items (also referred to as ‘burden’) needed to asses physical functioning and pain was compared between the PROMIS CAT, PROMIS short forms and the legacy PROMs.

##### Range of scores

Per measurement instrument, the percentage of patients with the minimal and maximum possible score were described.

### Statistical analysis

#### Reliability

##### Test-retest reliability

The ICC was calculated using a two-way random-effects model for absolute agreement: $\:ICC\:agreement=\frac{{\sigma\:}_{p}^{2}}{{\sigma\:}_{p}^{2}+{\sigma\:}_{m}^{2}+{\sigma\:}_{e}^{2}}$, whereby $\:{\sigma\:}_{p}^{2}$ is the variation between patients, $\:{\sigma\:}_{m}^{2}$ is the variation between measurements and $\:{\sigma\:}_{e}^{2}$ is random error variance. Test-retest reliability was considered sufficient if ICC ≥ 0.70 [16].

##### Measurement precision

The SEM for the legacy PROMs was calculated from the formula: $\:SEMagreement=\:\sqrt{{{\sigma\:}^{2}}_{m}\:{{+\:\sigma\:}^{2}}_{e}}$. Focusing on the absolute agreement, the variation between measurements (indicating systematic differences) is also considered error variance. The SEM (SE(T-score)) was provided for each patient score automatically when using PROMIS CAT software. For interpretation purposes, the mean and range of the SEM values were calculated and presented. There is no widely accepted method to compare the SEM or SDC of measurement instruments with different underlying theories (CTT versus IRT), since they have different scales. Therefore, the mean and the range presented can be used to interpret the corresponding measurement instrument and to compare measurement instruments on the same scale.

##### Smallest detectable change

The SDC is calculated as $\:SDC=\:1.96\:\text{*}\:\surd\:2\:\text{*}\:\text{S}\text{E}\text{M}$. For PROMs that use IRT-based scoring, the individual SEM of the test T-score and the individual SEM of the re-test T-score were used $\:(SDC=\:1.96*\sqrt{S{E}_{1}^{2}+S{E}_{2}^{2}})$. For traditional PROMs this will result in one SDC value (because there is only one SEM) per PROM, while for PROMs that use IRT-based scoring, this will result in a different SDC for each patient. Therefore, the mean and the range (T-scores) are presented per measurement instrument.

#### Validity

##### Construct validity

To assess construct validity, Pearson’s correlations were calculated between the PROMIS CAT and short forms, and the legacy PROMs. A matrix with all predefined hypotheses, resulting in 91 unique hypotheses, is presented in Supplemental Table 1. Construct validity was considered sufficient if ≥ 75% of the results was in accordance with the hypotheses.

## Results

**Fig. 1:**
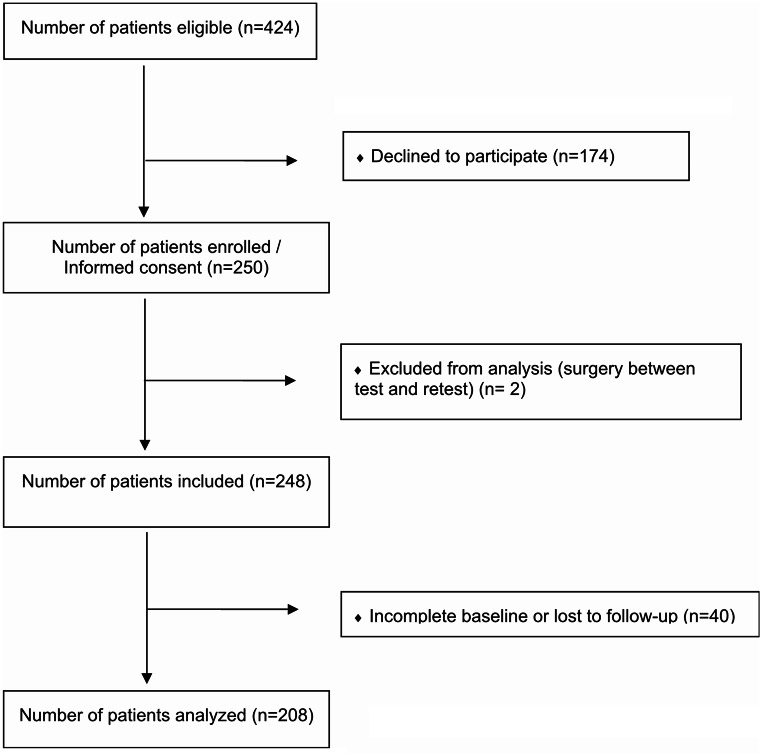
Flowchart of inclusion

In total, 208 patients were included in the analyses (Fig. 1). The mean age of the patients was 67.6 years, 62.8% were female (*n* = 130). The mean time-interval between test and retest was 8 days (SD 2). The mean score, standard deviation and range per measurement instrument at different time points can be found in Supplemental Table 2.

### Reliability

#### Test-retest reliability

All PROMIS CATs, PROMIS short forms and legacy instruments showed evidence of sufficient test-retest reliability (range ICC: 0.83–0.96, Table 2).

#### Measurement precision

**Fig. 2:**
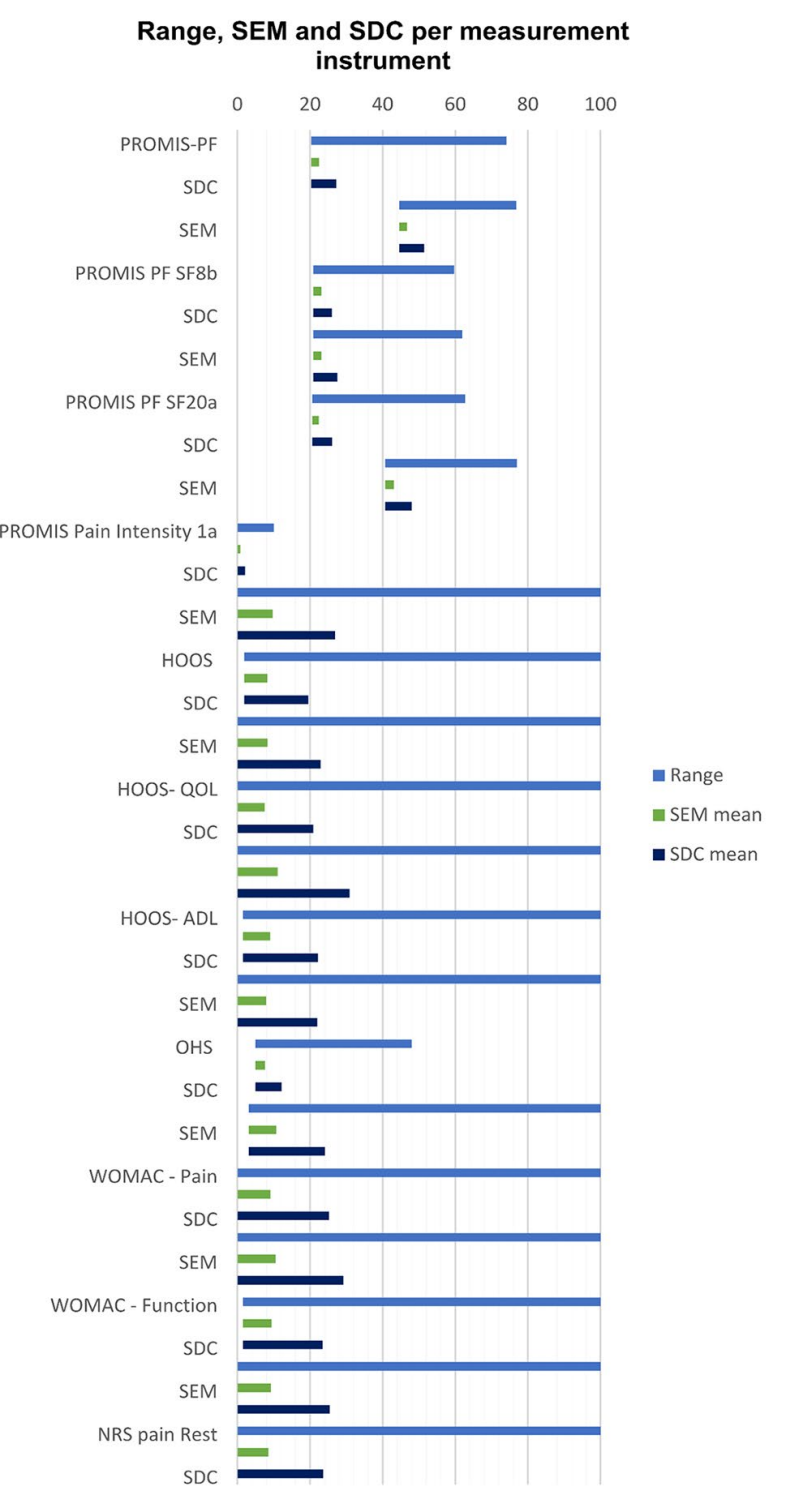
Range, SEM and SDC per measurement instrument

The mean SEM of PROMIS CAT and short forms was 1.8–2.2 on the T-score scale (observed score range 20.3–77; Table 2). The SEM of PROMIS Pain intensity was 0.8 (score range 0–10). The SEM of the legacy instruments varied between 6.3 and 11.1 of legacy instruments with a score range 0–100, and was 2.6 for the OHS (observed score range 5–48; Table 2). The possible range of the instruments can be found in Table 1, the range of the observed scores are presented in Table 2. The distribution of the scores, the SEM and the SDC are presented in Fig. 2.

#### Smallest detectable change

Table 2 gives details of the smallest detectable change of all PROMIS CATs, short forms and legacy instruments. The value of the SDC of PROMIS instruments was 2.1–7.3 T-score points (observed score range 20.3–77). The SDC of PROMIS Pain Intensity was 2.1 (score range 0–10). The SDC of the legacy instruments varied between 17.6 and 30.9 of legacy instruments with a score range 0–100. The SDC of the OHS was 7.2 (observed score range 5–48).

### Validity

#### Construct validity

The construct validity of PROMIS CAT and short forms measuring Physical Function were sufficient (92.3–100% of the results were in accordance with the hypotheses). The construct validity of the PROMIS Pain Intensity single item and PROMIS-PI were also found sufficient (both 92.3% of the results were in accordance with the hypotheses). The construct validity of PROMIS short form measuring Pain Interference was found insufficient (69.2% of the results were in accordance with the hypotheses) (Supplemental material Table 3).

### Interpretability

#### Burden

The number of items administered per measurement instrument are presented in Table 2. The burden of PROMIS-PF and PROMIS-PI was smaller than PROMIS short forms (4.8–5.2 versus 8–20 items). The burden of legacy instruments measuring physical functioning varied between 5 and 40 items.

#### Range of scores

Table 2 shows the percentage of patients with the minimal and maximum score per measurement instrument. Witch exception of the PROMIS Pain Interference SF8a, all measurement instruments measuring pain had a considerable percentage of patients with a minimal or maximum score (13–18% of the scores of PROMIS instruments, 15–29% of the scores of legacy instruments). None of the patients had the minimum or maximum value on the PROMIS short forms measuring Physical Function. Less than 1% of the patients had a maximum score on the PROMIS-PF.

## Discussion

The aim of this study was to determine if PROMIS CATs and short forms overcome the limitations of the legacy PROMs, by investigating the reliability, measurement precision, smallest detectable change, and burden of PROMIS Physical Function and Pain Interference CATs and short forms, and PROMIS Pain intensity, head-to-head to legacy PROMs in patients undergoing THA.

A clinically relevant finding is that PROMIS CATs are less burdensome with an equal reliability compared to legacy instruments in patients undergoing THA. Furthermore, this study reported on the SDC of many frequently used measurement instruments for patients undergoing THA. These SDC values per measurement instrument can be used as a guide to select a PROM with low measurement error, or as cut off values in the outpatient clinic to determine if it is likely that a patient has changed as result of the treatment.

This study faced methodological challenges in comparing the SEM and SDC between PROMIS and legacy instruments. The SEM and SDC can be used to interpret the measurement error of the measurement instruments and to compare measurement error of measurement instruments on the same scale. Although the absolute (mean) values of the SDC of PROMIS instruments were smaller than those of the legacy instruments, they cannot be directly compared, since measurement instruments have different scales (Fig. 2.). The SDC is a value that represents the change that can be detected with 95% confidence on the scale of the corresponding measurement instrument. However, scales differ in unit of measurement (score on a specific legacy instrument or T-score), range and level of measurement (ordinal versus interval). Legacy instruments are developed using CTT (in which each item contributes equal to the score) and PROMIS measurement instruments using IRT (each item has its own difficulty and a weighted score is used). IRT implies that PROMIS instruments have equal intervals between values (i.e., interval scale) and legacy instruments don’t (ordinal scale). To our knowledge, there is no consensus on how to address this problem. Several methods have been used in the literature to bypass this problem. One approach is to express the scores of different measurement instruments on the same IRT scale [24]. However, this method does not take into account that legacy instruments are not developed using IRT modelling. Other authors compared the percentage improved patients beyond measurement error, according to PROMIS and according to the legacy instruments [17]. Another solution would be to compare only ICC values, which relate the measurement error to the variation in scores. ICC values of the PROMIS measures were mostly higher than those of the legacy instruments. Because of the mentioned difficulties, this study presents the values per measurement instrument, accompanied with corresponding scales. More research is needed to determine the best approach to compare the measurement error of CTT-based and IRT-based instruments.

It should be noted that a lower CAT SE (SE 2.2, comparable to a reliability of 0.95) was used as stopping rule than the standard (SE 3.0, comparable to a reliability of 0.90). More reliable outcome scores can ensure more accurate individual patient monitoring, improve reliability of study results and can contribute to increase the use of patient reported outcome measures in the consultation room [18]. However, by using this setting it is presumable that the burden of the CATs increase (although in this study they were still lowest of all measurement instruments).

This study found that the PROMIS CAT and SF measuring Pain Interference were highly correlated with the comparator instruments measuring physical functioning in this patient population (resp. Pearson’s *r* =.82; 0.87). These correlations were even higher than the correlations with legacy instruments measuring pain. High correlations between PROMIS Physical Function and Pain Interference have also been found in previous studies [11, 22, 29], especially in patients suffering pain. It could be argued that for patients with pain these constructs are very similar. Because of this overlap in these constructs, it could be argued that there is no additional value measuring both in these patients. It could also be hypothesized that the construct pain is not relevant for all patient at every time moment, since most instruments measuring pain, had a considerable percentage of minimal or maximal possible scores, probably caused by the absence of pain post THA.

A possibly important aspect for THA patients when selecting the most suitable PROM for clinical practice is burden. Moreover, a smaller burden leads to less data storage, with subsequent reduction of the carbon footprint. A further reduction of the amount of data collected can be achieved by using PROMIS CAT and short forms, since these measurement instruments are generic and therefore the same PROMs can be used for multiple diagnoses.

When investigating alternatives for measuring physical functioning, the PROMIS-PF is less burdensome, has a wider measurement range (reducing floor/ceiling effects with more relevant questions) and almost no minimal or maximal possible scores, with an equal reliability compared to legacy instruments. When preferring a PROMIS Physical Function short form instead of PROMIS CAT, the 8-item PROMIS-PF SF 8b does not have a higher SEM or SDC than a short form containing more items (PF10a or PF20a). Furthermore, the 20-item PROMIS PF short form seems to add very little in score range beyond the PF 8b. Therefore, we recommend using the PROMIS-PF SF8b instead of PF10a or PF20A to reduce burden while obtaining an equal reliability and scoring range.

Regarding the construct pain, the PROMIS Pain Intensity 1a seems to be comparable to the legacy numeric rating scales measuring pain at rest and pain during activity in terms of burden, reliability and SDC. To facilitate the choice of an outcome measure, future research must focus on the minimally important change (MIC) and responsiveness of the different measures.

## Conclusion

The PROMIS-PF is a less burdensome alternative, with a wider measurement range (reducing floor/ceiling effects with more relevant questions) and almost no minimal or maximal possible scores, with an equal reliability, compared to legacy instruments measuring physical functioning in patients undergoing THA. The PROMIS Pain Intensity 1a seems to be comparable to the legacy numeric rating scales measuring pain at rest and pain during activity in terms of burden, reliability, and SDC. Measuring the construct Pain Interference may not have additional value to measuring physical function in patients undergoing THA. The SDC and SEM of many frequently used measurement instruments presented in this study can be used as a guide to select a PROM, or as cut off values in the outpatient clinic to determine if it is likely that a patient has changed as result of the treatment.

We want to thank Ariena Rasker for her contributions to the development of the design of the study and the data collection at the OLVG.

## Electronic supplementary material

Below is the link to the electronic supplementary material.

Supplementary Material 1

## Data Availability

The datasets used and/or analysed during the current study are available from the corresponding author on reasonable request.
