# Supplementary material for: Assessing the measurement properties of PROMIS Computer Adaptive Tests, short forms and legacy patient reported outcome measures in patients undergoing total hip arthroplasty
Source: J Patient Rep Outcomes. 2024 Oct 21;8:121. doi: 10.1186/s41687-024-00799-5 (PMC11493881; doi:10.1186/s41687-024-00799-5)
Supplement: Supplementary file 1 — Supplementary Material 1 [file 41687_2024_799_MOESM1_ESM.docx]

**Supplemental Table 1. Predefined hypotheses: expected correlations between the PROMIS CAT and PROMIS short forms Physical Function and Pain Interference, PROMIS single item Pain Intensity and legacy instruments**

|  | **Physical functioning** | | | | | |  |  | **Pain** | | |  | **Other domains** | | |
| --- | --- | --- | --- | --- | --- | --- | --- | --- | --- | --- | --- | --- | --- | --- | --- |
| **PROMIS** | **OHS** | **HOOS-PS** | **HOOS ADL** | **HOOS sport** | **HOOS** | **WOMAC function** | **MEAN** | **NRS pain activity** | **NRS pain rest** | **WOMAC pain** | **HOOS pain** | **MEAN** | **HOOS symptoms** | **WOMAC stiffness** | **HOOS QOL** |
| **PROMIS-PF** | A | A | A | A | A | A | $\bar{\boldsymbol{x}}$ | B | B | B | B |  | B | B | B |
| **PROMIS-PI** | A | A | A | A | A | A | $\bar{\boldsymbol{x}}$ | A | A | A | A | $\bar{\boldsymbol{x}}$ | B | B | B |
| **PROMIS-PF SF8b** | A | A | A | A | A | A | $\bar{\boldsymbol{x}}$ | B | B | B | B |  | B | B | B |
| **PROMIS-PF SF10a** | A | A | A | A | A | A | $\bar{\boldsymbol{x}}$ | B | B | B | B |  | B | B | B |
| **PROMIS-PF SF20a** | A | A | A | A | A | A | $\bar{\boldsymbol{x}}$ | B | B | B | B |  | B | B | B |
| **PROMIS-PI SF8a** | A | A | A | A | A | A | $\bar{\boldsymbol{x}}$ | A | A | A | A | $\bar{\boldsymbol{x}}$ | B | B | B |
| **PROMIS Pain intensity 1a** | B | B | B | B | B | B |  | A | A | A | A | $\bar{\boldsymbol{x}}$ | B | B | B |

*All correlations of PROMIS measurement instruments with the legacy measures were calculated. Measurement instruments measuring the same construct (e.g. PROMIS CAT Physical Function with WOMAC function subscale) were expected to have a Pearson’s correlation >0.7 (hypotheses A). The mean of these correlations was calculated per PROMIS measure (column ‘Mean’,* $\bar{\boldsymbol{x}}$*). Measurement instruments measuring related but different constructs were expected to have lower Pearson’s correlation (hypotheses B) than the mean correlation of PROMs measuring the same construct (*$\bar{\boldsymbol{x}}$***)*** *(e.g. PROMIS CAT Physical Function correlation with NRS pain activity would be lower than the mean correlation of PROMIS CAT Physical Function with legacy instruments measuring physical function)*

**Supplemental Table 2. Interpretability: average scores, range, presented as mean (SD; range).**

|  | Pre-surgery (n=118) |  | 3 months post-surgery (n=38) |  | 6 months post-surgery (n=24) |  | 12 months post-surgery (n=28) |  |
| --- | --- | --- | --- | --- | --- | --- | --- | --- |
|  | Baseline | Retest | Baseline | Retest | Baseline | Retest | Baseline | Retest |
| PROMIS-PF | 36.1(5.7; 20.3-51.6) | 36.2(5.8; 22.2-55.2) | 44.3(7.5; 25.2-61.6) | 45.6(6.9; 25.2-56.2) | 45(8.2; 27.8-57.6) | 44.5(7.3; 27.5-57.6) | 50.1(9.0; 32.6-74.1) | 47.5(9.3; 21.9-63.9) |
| PROMIS-PI | 65.5(5.5; 49.2-77.5) | 65.2(5.9; 48.2-76.8) | 56.2(8.7; 44.6-76) | 55.6(9; 44.6-73.1) | 54.4(8.7;44.6-73) | 54.2(8.4; 44.6-75.4) | 55(8.3; 44.6-69.2) | 56(9.3; 44.6-74.2) |
| PROMIS-PF SF8b | 34.6(5.8; 20.9-59.7) | 34.6(5.7; 20.9-59.7) | 43.1(8.6; 20.9-59.7) | 44.9(8.6; 20.9-59.7) | 44.7(8.8; 27.9-59.7) | 44.1(8; 30.1-59.7) | 50.7(9.1; 30.1-59.7) | 49.7(9.1; 30.1-59.7) |
| PROMIS-PF SF10a | 34.3(5.2; 20.9-53.4) | 34(5.6; 20.9-51.2) | 42.3(8.1; 26.9-61.9) | 44.6(7.7; 32.5-61.9) | 45.3(26-61.9) | 44.7(8.1; 27.7-61.9) | 49.8(9; 31.8-61.9) | 49(9.9; 26-61.9) |
| PROMIS-PF SF20a | 34.9(4.9;22.2-49.2) | 34.2(5.5; 20.6-48.3) | 43.1(7.6; 27.9-62.7) | 44.9(7.6;29.2-62.7) | 45.2(8; 30-62.7) | 44.6(7.8; 31.2-62.7) | 49.3(9; 32.4-62.7) | 48.8(9.9; 25.8-62.7) |
| PROMIS-PI SF8a | 63.6(5.6; 40.7-73.5) | 63.8(6.2; 40.7-77) | 51.7(9.3; 40.7-77) | 50.2(9.2; 40.7-71) | 48.5(10.3; 40.7-72.1) | 49.7(9.4; 40.7-69.2) | 47(8.6; 40.7-66.9) | 47.5(9; 40.7-66.9) |
| PROMIS Pain intensity 1a | 6.6(1.9; 0-10) | 6.8(1.8; 0-10) | 2.2(2.7; 0-9) | 2(2.8; 0-8) | 2.2(2.7; 0-9) | 2.61(2.7; 0-8) | 1.6(2.5; 0-8) | 1.9(2.8; 0-9) |
| HOOS | 33.2(16.4; 8.9-94.4) | 37.1(17.2; 1.9-91.3) | 77.2(19; 30-98.8) | 79(18.6; 26.9-99.4) | 81.8(17.9; 38.8-98.8) | 76(22.8; 24.4-98.1) | 89(14.3; 47.5-100) | 88.2(14.5; 55-100) |
| HOOS-PS | 46.1(17; 0-82.4) | 51(19.1; 4.6-100) | 20.2(15.4; 0-61.6) | 20.4(18;0-82) | 15.1(16.3; 0-67.9) | 18.8(17.1; 0-61.6) | 8.2(10.3; 0-30.4) | 10.4(13.1; 0-46.1) |
| HOOS-Symptoms | 40(19.4; 5-95) | 38.6(17.7; 0-90) | 76.6(18; 35-100) | 78.1(16.9;35-100) | 80(21.2; 35-100) | 75.9(23.1; 25-100) | 87.5(16.7; 50-100) | 85(18.5; 35-100) |
| HOOS-QOL | 23.9(18.8; 0-93.8) | 24.2(19.1; 0-93.8) | 67.4(24.8; 6.3-100) | 69.3(24.5; 6.25-100) | 77.2(23.7; 25-100) | 73.6(25.6; 12.5-100) | 82.4(24.9; 0-100) | 82.8(23.5; 18.8-100) |
| HOOS-Sport/Recr | 26.6(19.4; 0-87.5) | 23(19.7; 0-87.5) | 58.6(28.4; 0-100) | 62.9(26.6; 0-100) | 73.9(22.2; 25-100) | 66.3(27.6; 0-100) | 82.6(20; 43.8-100) | 79.9(24.9; 12.5-100) |
| HOOS-ADL | 44.8(17.7; 8.8-98.5) | 41.6(18.8; 1.5-97.1) | 80.5(18.9; 29.4-100) | 81.5(19; 23.5-100) | 83.8(16.5; 50-100) | 77.2(25; 25-100) | 91.1(12.7; 47.1-100) | 91.1(12; 64.7-100) |
| HOOS-Pain | 40.2(18.1; 5-100) | 39.6(17.7; 0-90) | 83.5(19; 35-100) | 85.4(17; 40-100) | 84.2(21.7; 27.5-100) | 78.8(24.2; 25-100) | 91.3(13.8; 47.5-100) | 90.4(13.7; 52.5-100) |
| OHS | 23.4(8.8; 5-47) | 22.6(9.6; 3-47) | 38.8(7.9; 14-48) | 39.7(8; 14-48) | 40.4(8.6; 10-48) | 39.6(9.9; 9-48) | 43.8(6.3; 19-48) | 43.1(8.3; 8-48) |
| WOMAC | 44.4(17.5; 9.4-99) | 42.3(18.5; 3.1-96.9) | 80.8(18.8; 31.3-100) | 82.1(18.2; 26-100) | 83.5(17.8; 40.6-99) | 77.5(24.5; 26-99) | 90.8(12.8; 47.9-100) | 90.2(12.5; 64.6-100) |
| WOMAC-Pain | 45.8(20.4; 0-100) | 45.2(18.8; 0-100) | 87(18.8; 35-100) | 87.9(16.2; 35-100) | 85.2(23.8; 25-100) | 80.7(24.8; 25-100) | 92.9(12; 55-100) | 91.6(11.3; 65-100) |
| WOMAC-Stiffness | 37.2(20.5; 0-100) | 37.4(19.2; 0-87.5) | 68.4(26.8; 0-100) | 68.8(24.7; 0-100) | 76.6(27.5; 0-100) | 72.3(26.4; 12.5-100) | 83.5(22.1; 25-100) | 79.5(26.2; 0-100) |
| WOMAC-Function | 44.8(17.7; 8.8-98.5) | 41.6(18.8; 1.5-97) | 80.5(18.9; 29.4-100) | 81.5(19; 23.5-100) | 83.8(16.5; 50-100) | 77.2(25; 25-100) | 91.1(12.7; 47-100) | 91.1(12; 64.7-100) |
| NRS Pain Activity | 73.4(18.1; 0-100) | 72.2(19.2; 0-100) | 24.5(27.9; 0-80) | 19.4(23.8; 0-80) | 24.5(27.9; 0-80) | 24.4(28.1; 0-80) | 12.14(19.1; 0-70) | 11.8(21.6; 0-90) |
| NRS Pain Rest | 53.7(23.3; 0-100) | 54.3(22.9; 0-100) | 11.6(16.7; 0-50) | 11.9(18.5; 0-70) | 15.2(22.9; 0-90) | 15.2(26.6; 0-90) | 8.6(19.4; 0-70) | 8.6(18.6; 0-70) |

*Abbreviations: SD= standard deviation; n= number of patients; CI= confidence interval; SDC= smallest detectable change; QOL= quality of life; Sport/Recr = sports/recreation; ADL = activities of daily living, NRS = numeric rating scale*

**Supplemental Table 3. Pearson’s r for correlations between PROMIS CATs and short forms and legacy instruments (n=208).**

|  | PF |  |  |  |  |  | Pain |  |  |  |  | Other |  |  |  | % according to hypotheses |
| --- | --- | --- | --- | --- | --- | --- | --- | --- | --- | --- | --- | --- | --- | --- | --- | --- |
| Measurement instrument | **OHS** | **HOOS-PS** | **HOOS ADL** | **WOMAC function** | **WOMAC total** | **MEAN** | **NRS pain activity** | **NRS pain rest** | **WOMAC pain** | **HOOS pain** | **MEAN** | **HOOS sport/rec** | **HOOS symptoms** | **WOMAC stiffness** | **HOOS QOL** |  |
| PROMIS-PF | .83 | -.74 | -.80 | .80 | .80 | **.79** | -.63 | -.72 | .76 | .79 |  | .77 | .77 | .74 | .78 | **92.3** |
| PROMIS-PI | -.85 | .76 | -.82 | -.82 | -.83 | **.82** | .70 | .78 | -.80 | -.83 | **.78** | -.75 | -.77 | -.73 | -.82 | **92.3** |
| PROMIS-PF SF8b | .84 | -.79 | .83 | .83 | .84 | **.83** | -.67 | -.76 | .78 | .81 |  | .83 | .80 | .79 | .82 | **92.3** |
| PROMIS-PF SF10a | .84 | -.81 | .84 | .84 | .83 | **.83** | -.70 | -.75 | .77 | .80 |  | .82 | .80 | .77 | .81 | **100** |
| PROMIS-PF SF20a | .85 | -.81 | .84 | .84 | .84 | **.84** | -.70 | -.76 | .78 | .80 |  | .82 | .81 | .78 | .81 | **100** |
| PROMIS-PI SF 8a | -.89 | .83 | -.88 | -.88 | -.89 | **.87** | .74 | .84 | -.86 | -.89 | **.83** | -.86 | -.86 | -.83 | -.89 | **69.2** |
| PROMIS Pain intensity 1a | -.86 | .81 | -.86 | -.86 | -.88 |  | .86 | .90 | -.86 | -.89 | **.88** | -.80 | -.84 | -.82 | -.86 | **92.3** |

*Green = hypotheses accepted; red = hypotheses rejected. PF = Physical Function*
